# Th17 cells in primary Sjögren’s syndrome negatively correlate with increased *Roseburia* and *Coprococcus*

**DOI:** 10.3389/fimmu.2022.974648

**Published:** 2022-10-05

**Authors:** Xiaohong Xin, Qian Wang, Jianbo Qing, Wenzhu Song, Yanni Gui, Xiaofeng Li, Yafeng Li

**Affiliations:** ^1^ Core Laboratory, Shanxi Provincial People’s Hospital (Fifth Hospital) of Shanxi Medical University, Taiyuan, China; ^2^ Institute of Nephrology, Peking University, Beijing, China; ^3^ Key Laboratory of Renal Disease, Ministry of Health of China, Beijing, China; ^4^ Shanxi Provincial Key Laboratory of Kidney Disease, Shanxi Provincial People’s Hospital (Fifth Hospital), Taiyuan, China; ^5^ Department of Nephrology, Shanxi Provincial People’s Hospital (Fifth Hospital) of Shanxi Medical University, Taiyuan, China; ^6^ School of Public Health, Shanxi Medical University, Taiyuan, China; ^7^ Department of Rheumatology and Clinical Immunology, Peking University First Hospital, Beijing, China; ^8^ Academy of Microbial Ecology, Shanxi Medical University, Taiyuan, China; ^9^ Department of Rheumatology, The Second Hospital of Shanxi Medical University, Taiyuan, China; ^10^ Key Laboratory of Cellular Physiology, Ministry of Education, Taiyuan, China

**Keywords:** primary Sjögren’s syndrome, gut microbiota, lymphocyte subpopulations, Th17 cells, Treg cells

## Abstract

**Background:**

Dysbiosis of the gut microbiota is closely related to chronic systemic inflammation and autoimmunity, playing an essential role in the pathogenesis of primary Sjögren’s syndrome (pSS). Abnormalities in the proportions of blood T lymphocyte subtype, that is Th17/Treg, were detected in pSS patients. We aimed to determine the associations between gut microbiota and Th17/Treg in pSS.

**Method:**

98 pSS patients and 105 healthy controls (NC) were enrolled between Dec 1, 2018, and Aug 31, 2019. The baseline information and clinical parameters on pSS patients and healthy controls were collected. 16S rRNA sequencing was performed to characterize the gut microbiome and identify gut microbes that are differentially abundant between patients and healthy controls. Lastly, associations between relative abundances of specific bacterial taxa in the gut and clinical outcome parameters were evaluated.

**Results:**

Patients with pSS show decreased gut microbial diversity and richness, decreased abundance of butyrate producing bacteria, such as *Roseburia* and *Coprococcus*, and increased abundance of other taxa, such as *Eubacterium rectale* and *Roseburia inulinivorans*. These bacteria are enriched with functions related to glycolytic and lipogenic, energy, substance, galactose, pentose metabolism pathways and glucuronate interconversions, decreased with functions related to peptidoglycan biosynthesis, pyrimidine metabolism pathways. An integrative analysis identified pSS-related specific bacterial taxa in the gut, for which the abundance of *Eubacterium rectale* is negatively correlated with Th17/Treg. Furthermore, the pathways of biosynthesis of secondary metabolites, biosynthesis of amino acids, peptidoglycan biosynthesis and pyrimidine, galactose, pentose, microbial metabolism in diverse environments, glyoxylate and dicarboxylate metabolism are associated with Treg or Th17/Treg.

**Conclusions:**

Primary Sjögren’s syndrome could lead to decreased gut microbial diversity and richness of intestinal flora in patients. The proportions of Th17 and Treg cells induced by microbiota were predictive pSS manifestations and accounted for the pSS severity.

## Introduction

Primary Sjögren’s syndrome(pSS) represents a common autoimmune condition characterized by a chronic immune response, contributing to inflammation and destruction of salivary and lacrimal glands (1). Although its etiology remains elusive, the pathogenesis is undoubtedly related to genetic factors, congenital and adaptive immune system abnormalities (2). Previously, B cell dysfunction is considered to play a key role in the initiation and development of pSS. However, B cell depletion did not show significant effects in pSS patients (3). Recently, the importance of T cells in the occurrence and development of pSS has been revealed, especially T helper 17 (Th17) cells and regulatory T (Treg) cells, which play a critical role in regulating the immune balance of systemic inflammation (4). Numerous murine models have demonstrated the implication of Th17/Treg cells imbalance in the disease induction. Th17/Treg cell imbalance can be attributed to the increased IL-6 level in the inflammatory environment. IL-6 synergizes with TGF-β to facilitate Th17 differentiation, while TGF-β in the absence of IL-6 promotes Treg differentiation (5). Treg cells in the lacrimal gland of C57BL/6.NOD.Aec1Aec2 mice are decreased, but Th17 cells and IL-17A expression are increased in comparison to those in the wild-type control mice in the early stage of pre-clinical disease (6). Consistently, transient depletion of Treg cells results in enhanced salivary gland infiltration in NOD mice (7). The effect of Th17/Treg cell imbalance on disease induction is further elucidated in thrombospondin-1 (TSP1)-depleted mice, an *in vivo* activator of latent TGF-β (8). These mice spontaneously present with ocular inflammation and dry eye symptoms, accompanied by elevated anti-SSA and anti-SSB antibodies (9). An increase in the number of splenic Th17 cells and an elevation in the levels of lacrimal IL-17 protein tend to coincide with a decrease in the number of splenic Treg cells in these mice (10). *In vivo* administration of TSP1 peptide can induce the formation of FoxP3þ Treg cells and reduce the number of Th17 cells in TSP1-knockout mice, thus alleviating disease symptoms (11). In NOD model mice in the presence of an altered MHC region (NOD.B10.H2b mice), these mice spontaneously develop ocular surface disease during aging. FoxP3þ Treg cells abnormally co-express transcription factors such as Tibet and RORgt to promote the production of IFN-γ and IL-17 in these aged mice. Moreover, Treg cells from aged NOD.B10.H2b mice possess lower suppressive capacity than Treg cells from young mice. Transferring CD4þCD25þ Treg cells from these aged mice to T and B cell-deficient (RAG1-deficient) animals can induce the phenotype of periductal inflammation in the lacrimal glands, which is similar to the effect of transferring CD4þCD25 T helper cells (12). To summarize, these findings of murine models illustrate that Treg cells can also acquire pro-inflammatory properties related to Th1 and Th17 cells, indicating that it is not only the enhanced pro-inflammatory properties of Th17 cells that promote disease.

Gut microbiota plays a role in maintaining the balance of immune responses between Treg and Th17 cells on the mucosal surface. The dysregulation of gut microbiota contributes to the development of rheumatic diseases (13) such as Behcet’s disease (14), systemic lupus erythematosus (15) and rheumatoid arthritis (16). Besides, numerous studies have presented altered gut microbiota compositions in pSS patients, such as depletions of *Faecalibacterium*, *Bacteroides*, *Parabacteroides* and *Prevotella* and richness of *Escherichia* and *Streptococcus* (17).. In addition, pSS patients are more likely to develop severe intestinal dysbiosis, which was related to clinical parameters of systemic disease activity and gastrointestinal inflammation (18). However, there are currently few studies to elucidate the underlying mechanisms by which gut microbiota affects the progression of pSS.

In this study, a total of 203 subjects were recruited, including 98 pSS patients and 105 healthy controls. We aim to comprehensively characterize the gut microbiome in pSS and explore the potential association between the gut microbiota and clinical parameters, especially with T cell homeostasis, through amplicon sequencing, machine learning (ML) and correlation analysis.

## Patients and methods

### Study participants and sample collection

From December 2018 to August 2019, patients were prospectively enrolled at the Second Hospital of Shanxi Medical University. The age-sex- and BMI-matched healthy volunteers were from routine physical examination in outpatient. All recruited pSS patients met the classification criteria of the 2016 American College of Rheumatology/European League Against Rheumatism (ACR/EULAR) (19). Participants with antibiotics treatment in the past two months or with previous gastrointestinal tract diseases were excluded in this study. Written informed consent was signed and the study was approved by the ethics committee of the Second Hospital of Shanxi Medical University (Ethics 64 Number: 2019-YX-107). Also, all fresh fecal samples were collected from study participants and then stored at -80°C. Peripheral blood of pSS patients was centrifuged (3000 g for 20 mins) and plasma and serum were obtained within one hour after collection for lymphocyte subpopulation and cytokine analysis.

### Clinical parameters

The baseline information, erythrocyte sedimentation rate (ESR), C-reactive protein (CRP), antibody (anti-Sjgren syndrome A antibody (SSA), anti-Sjgren syndrome B antibody (SSB), anti-Smith antibody (Sm), Rheumatoid factor (RF), Immunoglobin A (IgA), Immunoglobin G (IgG), Immunoglobin M (IgM) and complements in blood serum (complement 3 (C3), complement 4 (C4) were obtained from the medical records at admission and routine examinations. The levels of lymphocyte subpopulations in plasma were tested by using a modified method of flow cytometry, including T cells (CD3+ CD45+), B cells (CD19+ CD45+), CD4+T cells (CD3+ CD4+ CD45+), CD8+ T cells (CD3+ CD8+ CD45+), NK cells (CD16+ CD56+ CD45+), TBNK (T cells + B cells + NK cells), Th1 cells (CD4+ IL-2+), Th2 cells (CD4+ IL-4+), Th17 cells (CD4+ IL-17+), and Treg (CD4+ CD25+ FoxP3+). Besides, the serum levels of IL-2 (interleukin-2), IL-4 (interleukin-4), IL-6 (interleukin-6), IL-10 (interleukin-10), IL-17 (interleukin-17), Tumor Necrosis Factor-alpha (TNF-α), and Interferon γ (INF-γ) were detected using the flow cytometric bead array (CBA) (20). Moreover, the disease activity of pSS patients was assessed by EULAR Sjögren’s syndrome disease activity index (ESSDAI score) (21). We also collected subjective patient scores according to EULAR Sjögren’s syndrome patients reported index (ESSPRI score), which could be complemented with the ESSDAI score to help assess disease severity (22). Package **Table 1** (https://github.com/benjaminrich/table1) was used to draw the three-line table of clinical parameters.

**Table 1:** General characteristics of both control group (105 NC) and experimental group (98 pSS patients)^a^.

	NC (N = 105)	pSS (N = 98)	*P*-value
Age			0.115
Mean (SD)	52.8(9.45)	55.2 (11.9)	
Sex			0.994
Male	15 (14.3%)	13 (13.3%)	
Female	90 (85.7%)	85 (86.7%)	
BMI			0.419
Mean (SD)	23.4 (3.16)	23.1 (3.30)	

a chi-square test and t-test were employed for group comparison.

### Illumina sequencing and data processing

QIAamp PowerFecal DNA Kit (Qiagen) was applied to extract the microbial genome from approximately 250mg fecal samples based on the manufacturer’s instructions. Agarose gel electrophoresis and NanoDrop One (Thermo Fisher Scientific) were performed to examine the quality of DNA samples. Afterwards, DNA extracts were used to amplify V3–V4 hypervariable regions of the microbial 16S rRNA gene. Besides, FC magic beans Kit (enlighten) was used to purify and recover the product, which was quantified with Qubit 4.0 (Thermo Fisher Scientific). We diluted each sample to 4nM, mixing equal volumes of each cuvette, and denaturing them with sodium hydroxide. At least 5% of the Phix library was performed for a balance of the polymorphism, and each sample was sequenced on a Miseq PE300 (Illumina). Quality control for raw sequencing reads was performed *via* trimmomatic of low-quality bases from the reads 3’ end, and trimmed reads with a length<50 nt were excluded. Host (human) genome identification and removal were then employed *via* mapping into the human genome (hg38 build) with Bowtie2.

### Gut microbial diversity and differential gut microbiome

Software R (version 4.0.2) was employed for statistical analysis. Firstly, operational taxonomic units (OTUs) with a similarity cutoff of 99% (23) were selected using Userach10 for amplicon analysis. Afterwards, the genus-level alpha diversity indices were calculated with package vegan (version 2.5-6). Non-metric multidimensional scaling (NMDS) and Adonis analysis were also performed using package vegan and sample-to-sample similarities were weighted by Bray-Curtis distance. Furthermore, we employed Linear Discriminant Analysis (LDA) effect size (LEfSe) for the differential abundance of microorganisms to detect biomarker species.

### Construction of diagnostic model

Logistic Regression (LR) model (24) was applied for PSS and NC classification using the “logit function” in R. Receiver operating characteristic curve (ROC) and area under the curve (AUC) analysis were used for evaluation of the model performance, which was achieved using package pROC (version 1.16.2).

### Correlation analysis between gut microbiome and clinical parameters

Spearman’s correlation was conducted between clinical parameters and microorganisms using package psych (version 2.0.9).

### The function of gut microbiome and correlation analysis

Function prediction analysis based on bacterial biomarkers was performed on 16S, 18S or ITS sequencing data for prediction function abundance. Package picrust2 in R was used to obtain metagenome functions (25). Linear Discriminant Analysis (LDA) effect size (LEfSe) was used to assess the functional abundance for key metabolic pathways. Package psych was used to evaluate the correlation between microflora and pathway, as well as pathway and clinical parameters. All heatmaps were visualized by package pheatmap (version 1.0.12). Other visualizations were displayed using package ggplot2 (version 3.3.2), ggsci (version 2.9), and ggsignif (version 0.6.0).

### Statistical analysis

All analyses were performed using R studio (version 3.63). Normally distributed data were expressed as the mean ± SD and data with skewed distributions as the median and inter-quartile range. Wilcoxon rank test was used to compare continuous variables with skewed and normal distributions, respectively. Categorical variables in different groups were compared using the Chi-square test. Correlation between clinical parameters and microorganisms was evaluated using Spearman correlation analysis. P < 0.05 was considered to be statistically significant.

## Results

### Baseline characteristics and clinical profiles

The baseline characteristics of participants were listed in **Table 1** and **Supplementary Data 1**. The mean age was 55.2 and 52.8 years in pSS and NC group, respectively (*P=*0.115). There were 86.7% females in pSS group and 85.7% females in NC group (*P*=0.994). The mean BMI was 23.1 kg/m2 and 23.4 kg/m2 in pSS and NC group, respectively (*P*=0.419) (**Table 1**). In addition, pSS patients were further divided into three groups according to ESSDAI score (group1: inactive, ESSDAI score ≤ 5; group2: moderately active, 5<ESSDAI score ≤ 13; group3: severe active, ESSDAI score>13).

### Gut microbial diversity

**Figure 1:**
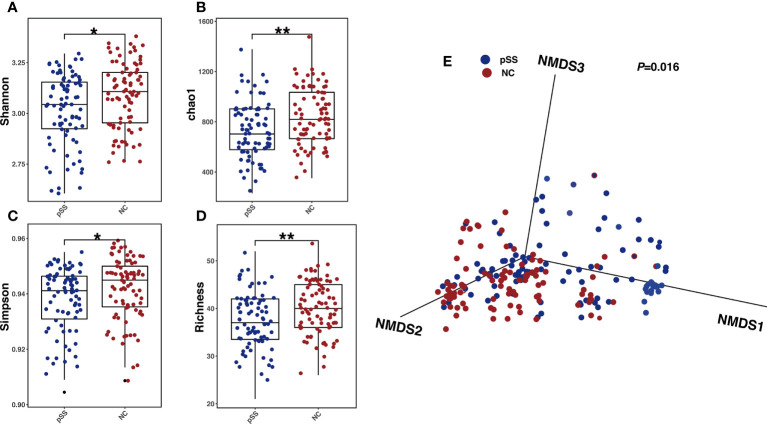
Gut microbial alterations in pSS patients compared with healthy controls. Comparison between Gut microbial profile of samples from healthy controls and pSS participants. **(A)** Shannon index between groups revealing microbial richness and particular evenness(alpha-diversity); *P*-values were calculated by Wilcoxon rank-sum test; **(B)** Simpson index levels of healthy controls and pSS participants; *P*-values were also calculated by Wilcoxon rank-sum test. **(C)** Richness of Gut microbial profile of samples in two groups with *P*-values calculated by Wilcoxon rank-sum test. **(D)** Chao 1 index and the number of species with *P*-values yielded from Wilcoxon rank-sum test.”**”: adjusted *P-*values “<0.01”,”*”:adjusted *P*-value “<0.05”. **(E)** NMDS analysis revealing β diversity based on Bray-Curtis Distance, with R^2^ and *p* value calculated from Adonis analysis.

Gut microbial composition was analyzed *via* amplicon sequencing of 203 fecal samples using Usearch10 with 108 species and 60 genera identified (**Supplementary Table 2**). The indices of Shannon, Simpson, chao1 and Richness in pSS patient samples were significantly lower than those of the NC group (*P*<0.01), indicating a lower bacterial diversity in pSS patients (**Figures 1A–D**). The nonmetric multidimensional scaling (NMDS) demonstrated that the gut microbiota compositions in the pSS and NC were clearly separated (**Figure 1E**) with different similarities weighted by Bray-Curtis distance (*P* =0.016). The above results highlighted significant alterations in gut microbes between pSS patients and NC.

### Differential gut microbiome

**Figure 2:**
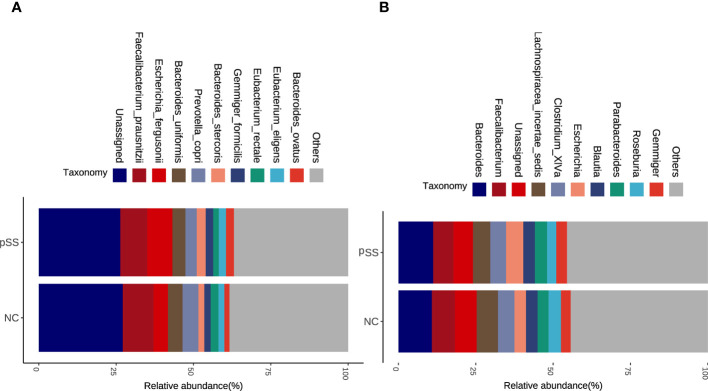
Altered microbial composition in pSS adjusting for potential confounding factors. Stacked bar plots of top ten abundant genera **(A)** and species **(B)** in pSS and healthy controls *via* gut microbial profiling.

**Figure 3:**
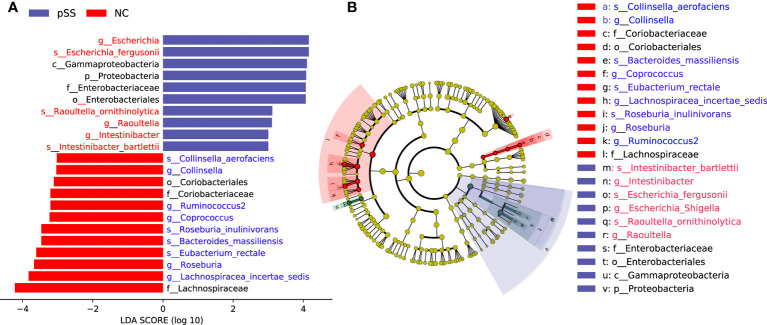
Identification of differential gut microbiota in pSS patients. Lefse analysis was performed to identify differentially abundant taxa, which are highlighted on the phylogenetic tree in cladogram format **(B)** and for which the LDA scores are shown **(A)**.

The analysis based on genus and species levels showed that pSS patients contained less beneficial bacteria and more pathogenic bacteria compared with the NC group (**Figures 2A, B**), with an LDA score >3 in pSS patients. In genus level, the following seven were more obvious, namely *Lachnospiracea incertae sedis, Coprococcus, Ruminococcus2, Intestinibacter, Escherichia, Collinsella, Raoultella*, and *Roseburia*. In species level, there were seven significantly different bacteria, including *Bacteroides massiliensis, Roseburia inulinivorans, Collinsella aerofaciens, Escherichia fergusonii, Eubacterium rectale, Intestinibacter bartlett*, and *Raoultella ornithinolytica* (**Figure 3A**). The evolutionary tree of all differential gut microbiomes was shown in **Figure 3B**.

### Identification of gut microbiome-derived signatures in diagnosing pSS

**Figure 4:**
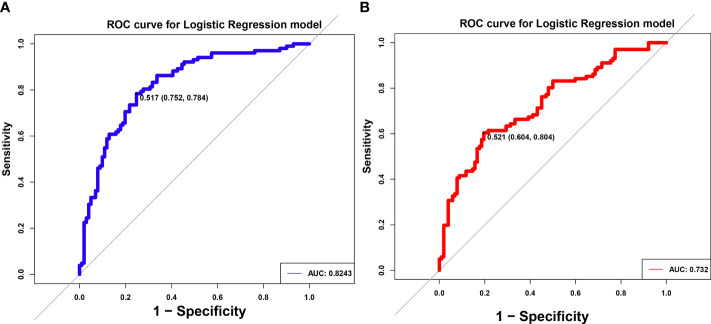
Identification of differential gut microbiota in pSS patients. ROC of logistic regression (LR) model using eight differential genus **(A)** and seven differential species **(B)**. The AUC were 0.8243 and 0.732, separately.

In order to explore whether species-level information alone can predict specific social relationships, a machine learning approach that utilized organism abundances was implemented. The LR model could distinguish pSS patients from NC by gut microbiome classified by 8-differential genera or 7-differential species. The AUC were 0.8243 (95%CI:0.752-0.784) and 0.732 (95%CI:0.804-0.604) respectively in the models, suggesting that gut microbiomes from different genera and species could serve as a good approach for auxiliary diagnosis for pSS (**Figures 4A, B**).

### Gut microbiome-derived signatures for pSS and their association with clinical parameters

**Figure 5:**
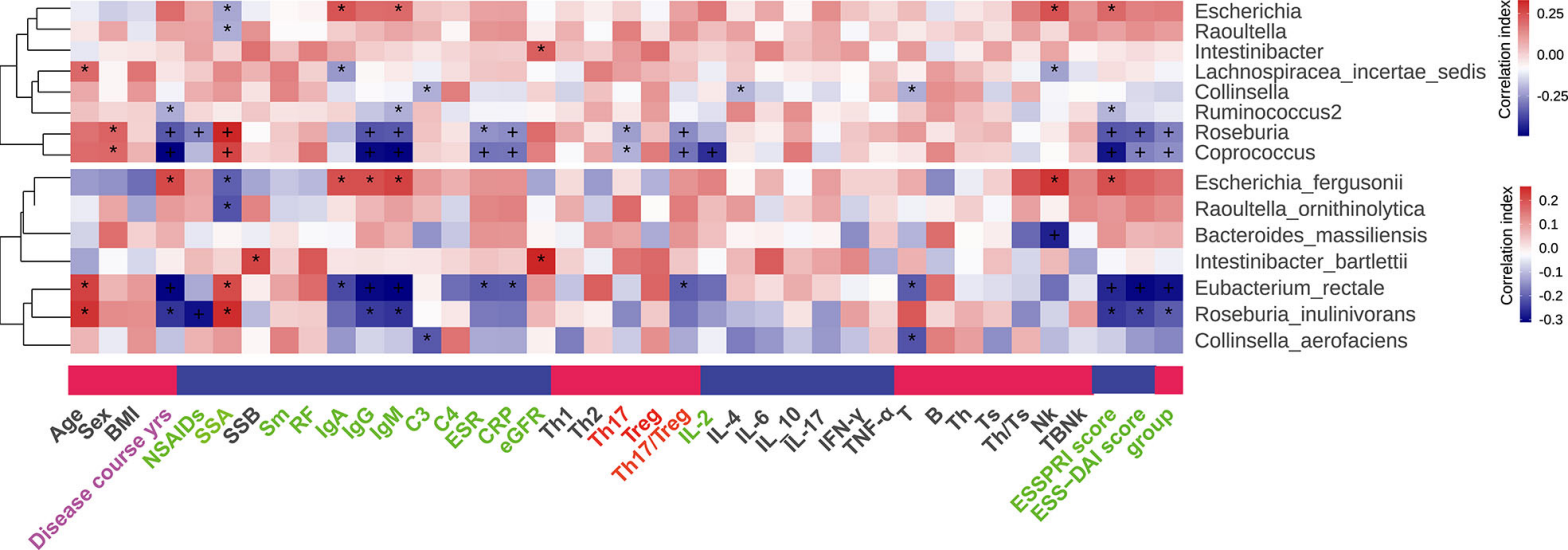
Correlation between differential gut microflora and clinical parameters, “+” and “*”, adjusted *p* values “<0.01” and “<0.05”.

The association between clinical parameters and gut microbiome was analyzed. In genus level, *Roseburia* and *Coprococcus* were positively correlated with the level of SSA, and negatively correlated with ESR, CRP, IgG, IgM, Th17, Treg, Th17/Treg, ESSPRI score, ESSDAI score in the group of pSS patients. Similarly, in species level, *Roseburia inulinivorans and Eubacterium rectale* were positively correlated with the level of SSA, and negatively correlated with ESR, CRP, IgG, IgM, ESSPRI score, ESSDAI score and groups of pSS patients. Particularly, *Eubacterium rectale* was negatively correlated with the ratio of Th17/Treg (**Figure 5**). Our results thus reveal a robust relationship at taxa-level between Th17/Treg and microbiome composition that is independent of other factors.

### Functional characterization of pSS microbiome

**Figure 6:**
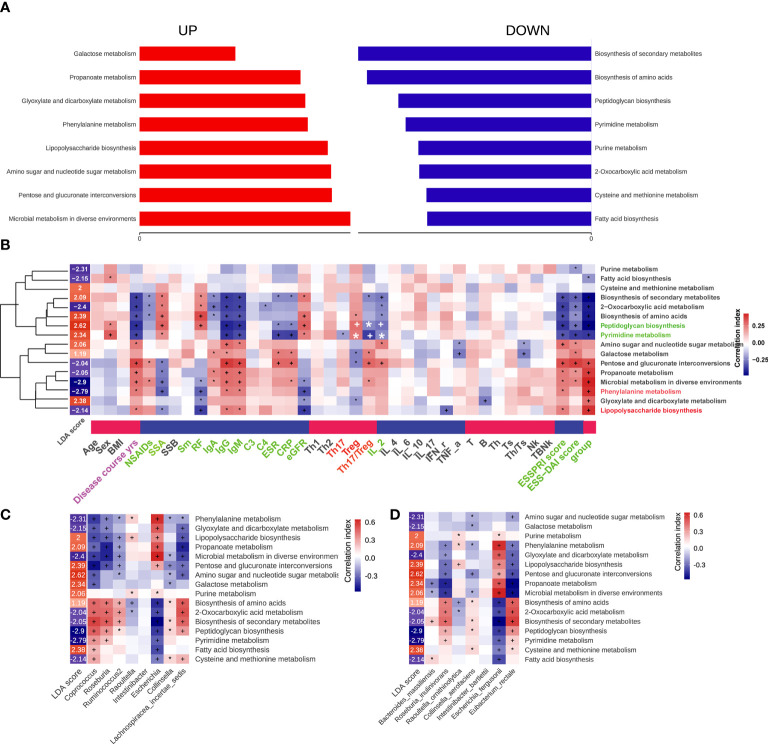
Functional characterization of pSS microbiome. The differential Kegg (Kyoto Encyclopedia of Genes and Genomes) functions of gut microbiota that predicted by package Picrust2 in R. **(A)** The correlation between differential pathways and clinical parameters. **(B)** The correlation between differential pathways and differential genus. **(C)** The correlation between differential pathways and differential species. “+” and “*”, adjusted *p* values “<0.01” and “<0.05”. **(D)** It was our writing mistake and we have already made some changes in the manuscript. The correlation between differential pathways and differential species. “+” and “*”, adjusted *p* values “<0.01” and “<0.05”.

Alterations of 16 altered pathways in pSS patients were identified *via* Picrust2, including eight increased pathways, such as microbial metabolism in diverse environments, Pentose and glucuronate interconversions, Amino sugar and nucleotide sugar metabolism, Lipopolysaccharide biosynthesis, Phenylalanine metabolism, Glyoxylate and dicarboxylate metabolism, Propanoate metabolism, and Galactose metabolism. Meanwhile, there were eight decreased pathways, including Fatty acid biosynthesis, Cysteine and methionine metabolism, 2-Oxocarboxylic acid metabolism, Purine metabolism, Pyrimidine metabolism, Peptidoglycan biosynthesis, Biosynthesis of amino acids and Biosynthesis of secondary metabolites (**Figure 6A**). In addition, correlations between 16 pathways and clinical features parameters were also assessed, of which 16 pathways were mainly related to disease course, antibody levels, ESSPRI score, ESSDAI score and groups of pSS patients. It’s worth noting that Biosynthesis of secondary metabolites, Biosynthesis of amino acids, Peptidoglycan biosynthesis, Pyrimidine metabolism, Galactose metabolism, Pentose and glucuronate interconversions, Microbial metabolism in diverse environments, Glyoxylate and dicarboxylate metabolism were associated with Treg or Th17/Treg (**Figure 6B**). Additionally, the correlation between the differential gut microbiome and the pathways were shown in **Figures 6C, D**. Interestingly, the presence of *Coprococcus* and *Eubacterium rectale* were involved in most of the altered pathways.

## Discussion

A growing body of experimental and clinical evidence has suggested that the occurrence of gut microbiota-related diseases is associated with immunopathogenesis (26, 27). Gut microbiota has been involved in elucidating the pathogenesis of considerable gut and systemic autoimmune diseases. Intestinal mucosa contains the largest population of immune cells and plays an indispensable role in innate and adaptive immunity (28). Gut microbiota and the intestinal barrier together maintain the balance of intestinal immune system through mutual coordination and interaction (29). The integrity of the gut barrier is important for constraining the microbiota to no longer trigger adaptive immune responses (30). Alterations in gut microbial composition not only make the immune responses irregular, impairing the intestinal mucosal barrier, but also lead to the increase in inflammatory cytokines and changes in the balance of various immune cells, further resulting in various chronic disorders (31).

pSS is a systemic autoimmune disease characterized by dryness of the mouth and eyes and lymphocyte infiltration in exocrine glands (32). Environmental and genetic factors contribute to the abnormal activation of autoimmune responses in pSS (33). Abnormal proliferation of B cells, excessive secretion of lymphocytosis and cytokines, abnormal distribution and transport of water molecule channel proteins in exocrine glands, as well as resistance to anti-M3R antibody-mediated inhibition of acetylcholine secretion in exocrine gland lead to systemic inflammation in pSS (34). The increase of immunoglobulins and various autoantibodies in the blood is considered the most significant characteristic of pSS, which might indicate that pSS is a typical B cell-induced autoimmune disease (35). However, glandular tissue in pSS is heavily infiltrated by T lymphocytes in the multiple phases of the disease (4), and B-cell depletion with rituximab also failed to improve pSS symptoms (36), suggesting that T cells also play an indispensable role in the pathogenesis of pSS.Th1 and Th2-derived cytokines such as IL-1, IL-6, IL-4, IL-10 TNF-α, and IFN-γ mediate the pathological damages in the exocrine gland (37, 38). In addition, Th17 cells may promote B cell activation by secreting cytokines such as IL17 (39), leading to increased levels of autoantibodies. Th17 polarization actively contributes to the development of pSS (40, 41). In contrast, Tregs are thought to have a countervailing suppressive effect on Th17 cell-mediated systemic inflammation. Depletion of Tregs in peripheral blood and tissues of pSS patients breaks the balance between Th17 and Treg, leading to active local immune responses that ultimately result in immune imbalance and systemic inflammation (42). A dynamic balance between Th17 cells and Tregs is of great significance for human health and maintaining Th17/Tregs homeostasis may be a central way to reduce systemic inflammation of pSS. It is worth mentioning that the gut microbiome is involved in the differentiation of self-reactive Th17 cells and Tregs (43, 44).

Growing experimental and clinical evidence has suggested that gut microbiome alterations play an indispensable role in the occurrence and development of pSS. In this study, the combined gut microbial markers of eight genera and seven species were accurately identified, with AUCs of 0.8243 and 0.732, respectively. The alterations of gut microbiota in pSS indicate it may be a potential therapeutic target for pSS. The correlation analysis between differential gut microbiota and clinical parameters of pSS patients demonstrated that gut microbiota was closely related to autoantibody levels, ESR, CRP, Th17, Treg, Th17/Treg, ESSPRI score, ESSDAI score. All these results confirm a potential link between gut microbiota and pSS.

Two prominent genera in the intestinal flora, *Roseburia* and *Coprococcus*, were significantly reduced in the intestines of pSS patients. Such decreases were related to the severity of the disease and the increase of Th17 cells. *Roseburia intestinalis* is a beneficial gut organism and plays an important role in regulating barrier homeostasis, immune cell activation, and cytokine release (45). Similarly, *Coprococcus* plays an anti-inflammatory role in a variety of diseases by modulating serum markers of inflammation (46). As reduced species, *Roseburia inulinivorans* and *Eubacterium rectale* were also associated with the activated inflammation of pSS. In addition, a study has also shown that *Eubacterium rectale* can regulate the systemic inflammation (47). Overall, *Roseburia* and *Eubacterium* play a critical role in the maintenance of human health (48). The reduction of beneficial bacteria leads to the pathogenesis of pSS, and Th 17 cell and Treg may represent the link between them.

Intestinal mucosa contains a large number of Th17 cells and Tregs, which are exposed to various foreign antigens and related to recruitment and differentiation of T-helper (Th) cells, as well as cytokine production (49){II, 2006 #409;CL, 2007 #410}. Th17 cells play a critical role in protecting mucosal surfaces against microbial pathogens and Tregs allow for restraining excessive inflammatory responses. The differentiation of Th17 cells and Treg is finely regulated by many factors, such as transforming growth factor-β(TGFβ) and IL-6 (50). Notably, the specific components and metabolites of the gut microbiome have been implicated in the production of proinflammatory cytokines and the differentiation of T cells (49). Although the precise mechanism of T cell differentiation regulated by gut microbiome remains unclear, several studies have indicated that microbe-derived short-chain fatty acids (SCFAs) may be involved in the Treg induction (51). A recent study demonstrates that butyrate is involved in Treg differentiation (23). In addition, glycolytic and lipogenic pathways were necessary for energy production of activated effector T-cells, which could regulate the balance between inflammation and immune homeostasis by favoring T cell differentiation toward Th17 cells or Tregs (52). Besides, gut microbiota and their metabolites may be associated with T cell differentiation through various metabolism pathways.

Hundreds of molecules synthesized by gut microbiota could influence host physiology (53), and the metabolic activity of gut microbiota is an important energy source for intestinal epithelial cells (54). *Coprococcus* and *Eubacterium rectale* were involved in pathways related to Tregs and Th17/Treg levels, such as increased galactose metabolism pathway and pentose and glucuronate interconversions, and decreased peptidoglycan biosynthesis pathway and pyrimidine metabolism. The change in metabolic activity of gut microbiota may be subjected to energy production of activated effector T-cells, which is closely related to the differentiation of Th17 cells and Tregs. Regulation of the Th17/Treg axis affects the balance between pro-inflammatory and anti-inflammatory mechanisms (55), and gut microbiota and its metabolites may play an indispensable role in this process.

## Conclusion

Gut microbiome in pSS patients is characterized by an altered richness and diversity, which may not solely be the consequence of pSS, but affects the occurrence and development of pSS. Also, the Th17/Treg axis is associated with gut microbiome in patients with pSS.

## Data availability statement

The original contributions presented in the study are included in the article/**Supplementary Material**. Further inquiries can be directed to the corresponding authors.

## Author contributions

XX Performed data analysis and wrote the draft. JQ was in charge of screening patients. XX and QW collected the samples and processed the samples. WS and YG modified the paper. XL and YL were responsible for the final review of the paper. All authors contributed to the article and approved the submitted version.

## Funding

This project was supported by the National Science Foundation of China (8217021,81870333),Returned Overseas Students Scientific Research Project Supported by Shanxi Scholarship of Council Of China(2020-183).

## Acknowledgments

We appreciate all the authors and patients participating in this study. We are indebted to those fecal donors in our study.

## Conflict of interest

The authors declare that the research was conducted in the absence of any commercial or financial relationships that could be construed as a potential conflict of interest.

## Publisher’s note

All claims expressed in this article are solely those of the authors and do not necessarily represent those of their affiliated organizations, or those of the publisher, the editors and the reviewers. Any product that may be evaluated in this article, or claim that may be made by its manufacturer, is not guaranteed or endorsed by the publisher.
